# Supplementary material for: Astragalus polysaccharides (PG2) Enhances the M1 Polarization of Macrophages, Functional Maturation of Dendritic Cells, and T Cell-Mediated Anticancer Immune Responses in Patients with Lung Cancer
Source: Nutrients. 2019 Sep 20;11(10):2264. doi: 10.3390/nu11102264 (PMC6836209; doi:10.3390/nu11102264)
Supplement: Supplementary file 1 [file nutrients-11-02264-s001.pdf]

# SUPPLEMENTARY INFORMATION

## Astragalus polysaccharides (PG2) enhances the M1 polarization of macrophages, functional maturation of dendritic cells, and T cell-mediated anticancer immune responses in patients with lung cancer

Oluwaseun Adebayo Bamodu[a,b#], Kuang-Tai Kuo[c,d#], Chun-Hua Wang[e,f], Wen-Chien Huang[g,h], Alexander T.H. Wu[i], Jo-Ting Tsai[j,k], Kang-Yun Lee[l], Chi-Tai Yeh[c,d,m*] and Liang-Shun Wang[a,b] *

[a] Division of Hematology & Oncology, Department of Medicine, Shuang Ho Hospital, Taipei Medical University, New Taipei City, Taiwan.

[b] Department of Medical Research and Education, Shuang Ho Hospital, Taipei Medical University, New Taipei City, Taiwan.

[c] Division of Thoracic Surgery, Department of Surgery, Shuang Ho Hospital, Taipei Medical University, New Taipei City, Taiwan.

[d] Division of Thoracic Surgery, Department of Surgery, School of Medicine, College of Medicine, Taipei Medical University, Taipei, Taiwan.

[e] Department of Dermatology, Taipei Tzu Chi Hospital, Buddhist Tzu Chi Medical Foundation, New Taipei City, Taiwan.

[f] School of Medicine, Buddhist Tzu Chi University, Hualien, Taiwan

[g] MacKay Medical College, Taipei, Taiwan.

[h] Division of Thoracic Surgery, Department of Surgery, MacKay Memorial Hospital, Taipei, Taiwan.

[i] The Ph.D. Program for Translational Medicine, College of Medical Science and Technology, Taipei Medical University, Taipei, Taiwan

[j] Department of Radiation Oncology, Shuang Ho Hospital, Taipei Medical University, New Taipei City, Taiwan.

[k] Department of Radiology, School of Medicine, College of Medicine, Taipei Medical University, Taipei, Taiwan.

[l] Division of Pulmonary Medicine, Department of Internal Medicine, Shuang Ho Hospital, Taipei Medical University, Taiwan.

[m] Department of Biotechnology and Pharmaceutical Technology, Yuanpei University of Medical Technology, Hsinchu City 30015, Taiwan

# These authors contributed equally to this work.
*Corresponding authors:

Dr. Chi-Tai Yeh
Department of Medical Research and Education, Shuang Ho Hospital, Taipei Medical University New Taipei City 23561, Taiwan; Tel: +886-2-2490088 ext. 8881; Fax: +886-2-2248-0900. E-mail addresses: ctyeh@s.tmu.edu.tw

Dr. Liang-Shun Wang

Division of Thoracic Surgery, Department of Surgery, Shuang Ho Hospital, Taipei Medical University, New Taipei City, Taiwan. Phone: 886-2-2490088 ext. 8885; FAX: 886-2-2248-0900. E-mail: wangls72269@yahoo.com.tw

**Working title:** PG2 mediates broad spectrum anticancer immunogenicity

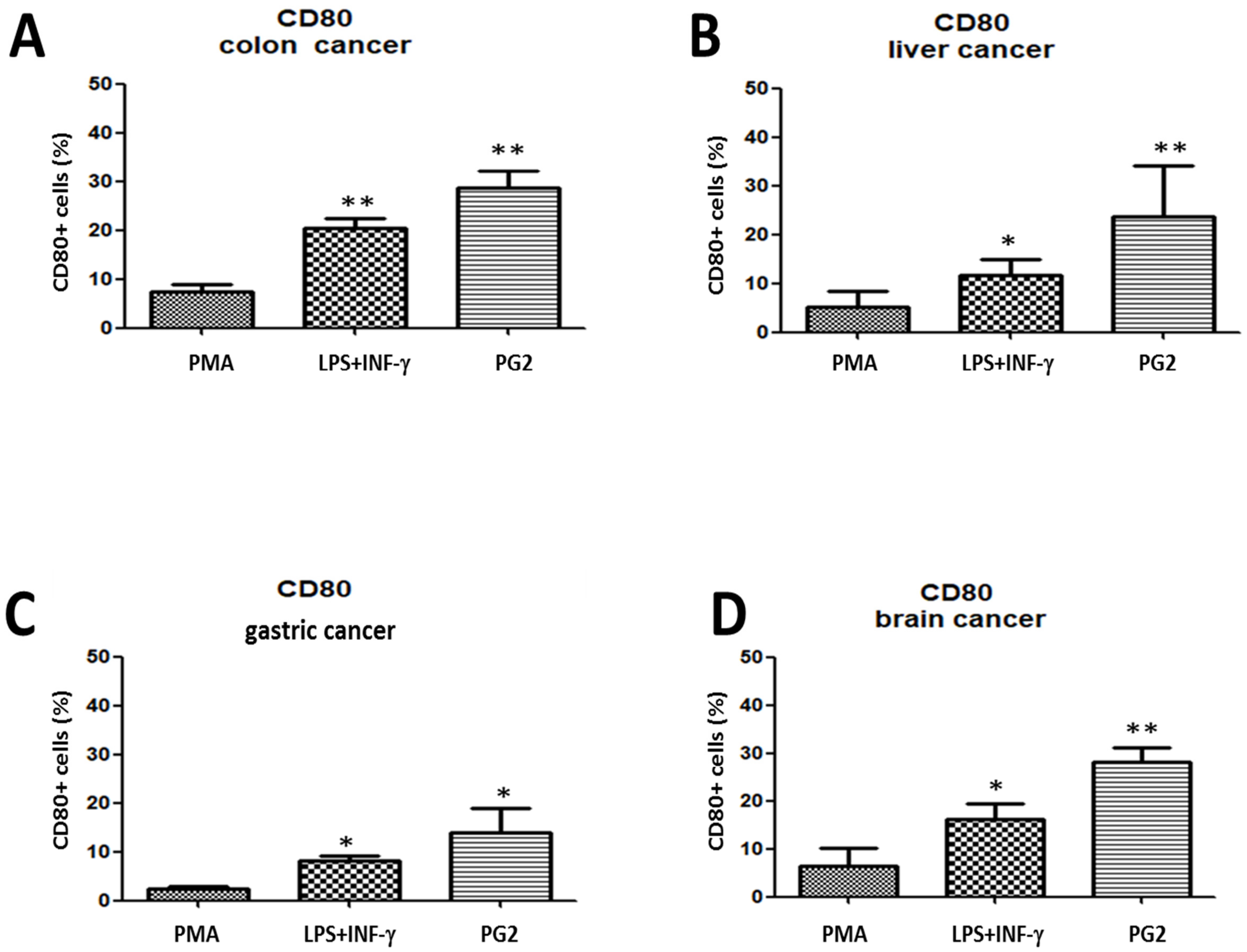


**Supplementary Figure S1.** PG2 enhances the production of CD80+ M1 macrophage-derived DCs from PBMCs of different type of cancer patients.

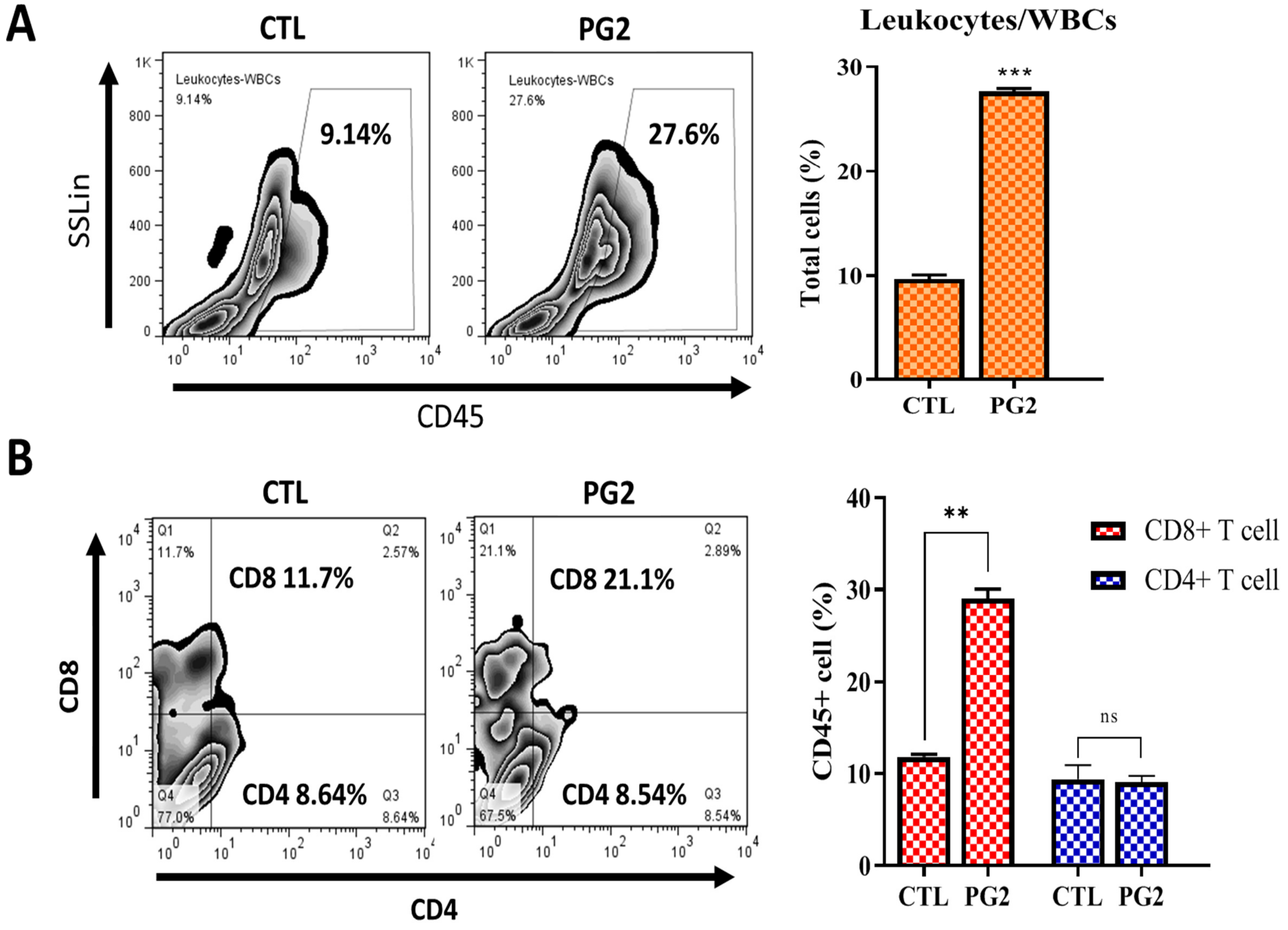


**Supplementary Figure S2.** PG2 treatment elicits significant increase in tumor infiltrating CD8+ T cells population. Representative flow cytometry data (left panel) and histograms (right panel) showing the effect of PG2 on the mean (A) CD45+ leukocyte, and (B) CD8+ or CD4+ T cell population in disassociated patient-derived cell suspensions compared to untreated control tumor samples. *p<0.05, **p<0.01, ***p<0.001, ns, not significant.

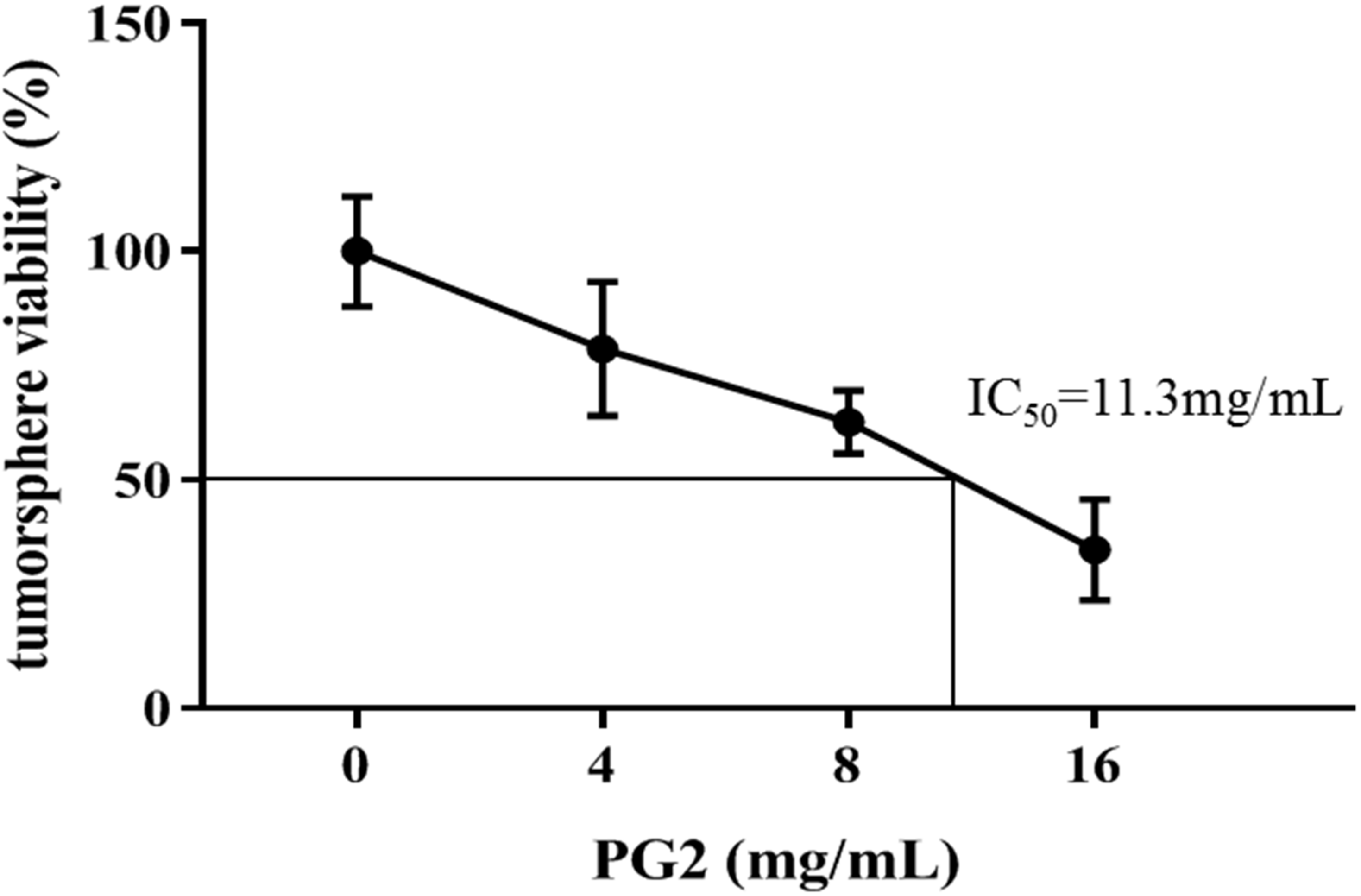


**Supplementary Figure S3.** PG2 suppressed the viability of tumorspheres derived from M2/H1229 co-culture system. Compared to the untreated control, PG2 concentration at the number of tumorspheres formed were reduced by 50% is indicated and used for dichotomization of PG2 dosage as high (16 mg/mL) or low (8 mg/mL).
